# Flow diversion of a dissecting PICA aneurysm

**DOI:** 10.3171/2022.7.FOCVID2247

**Published:** 2022-10-01

**Authors:** Tyler Lazaro, Viren Vasandani, Ariadna Robledo, Nisha Gadgil, Peter Kan

**Affiliations:** 1 Department of Neurosurgery, Baylor College of Medicine, Houston; and; 2 Department of Neurosurgery, University of Texas Medical Branch, Galveston, Texas

**Keywords:** flow diversion, Silk Vista Baby, cerebral aneurysm, dissecting aneurysm

## Abstract

A 47-year-old female with a history of a ruptured left posterior inferior cerebellar artery (PICA) aneurysm, status post coil embolization and retreatment for recurrence, presented with evidence of a recurrent dissecting PICA aneurysm. Given that these aneurysms are considered high risk and have a greater propensity for rupture than anterior circulation aneurysms, retreatment was recommended. With the patient’s strong preference for endovascular therapy, flow diversion with a Silk Vista Baby was performed. Given the low-profile design of the device, a radial artery approach and coaxial technique were used to deploy the flow diverter. The device was successfully placed, with complete obliteration of the aneurysm after 1 year.

The video can be found here: https://stream.cadmore.media/r10.3171/2022.7.FOCVID2247

## Transcript

Hello, I am lead author, Tyler Lazaro, from Baylor College of Medicine, and I will be discussing a case of flow diversion for a dissecting PICA aneurysm.

None of the contributing authors of this video have any financial disclosures.

### 0:33 Case Presentation.

Patient is a 47-year-old woman with a previously coiled ruptured left PICA aneurysm and subsequent recurrent left PICA aneurysm status post additional coiling, who presented for routine follow-up cerebral angiography.

### 0:47 Preprocedure Angiogram.

AP and lateral projections of a left vertebral artery injection are shown here, revealing a 2-mm fusiform dilation at the base of the previously coiled left PICA aneurysm, which is at the junction of the anterior and lateral medullary segments.

### 1:03 Diagnosis.

Thus, the diagnosis is a recurrent 2-mm dissecting left posterior inferior cerebellar artery aneurysm.

### 1:12 PICA Aneurysms.

A quick word on PICA aneurysms: They are considered high-risk aneurysms, with a greater propensity for rupture than anterior circulation aneurysms. They most often occur at the take-off from the vertebral artery or proximal segments of the PICA. In addition, the diameter of the PICA artery is quite diminutive, at approximately 1.8 mm, which tapers to a smaller diameter more distally. This has previously made endovascular intervention, particularly flow diversion, very challenging.

### 1:46 Dissecting PICA Aneurysms.

In our case, we are also dealing with a dissecting PICA aneurysm, which are rare, usually involve segments distal to p2, and carry an even higher risk of rupture.

### 1:58 Treatment Options.

Given the natural history of dissecting PICA aneurysms and the multiple recurrences in this patient, retreatment was recommended. With the availability of the Silk Vista Baby [Balt] in mind, parent vessel reconstruction with flow diversion was offered to the patient. Were this not an option, stenting or the use of standard flow diverters would be considered high risk for parent vessel thrombosis, given the narrow diameter of the more distal PICA segments as previously discussed, which would be a poor outcome in a patient with an unruptured recurrence and who is neurologically intact.

Moreover, while vessel deconstruction and revascularization with surgery was an option, there were several considerations that favored endovascular flow diversion in this case. For one, this patient had very small occipital arteries, making aneurysm trapping and occipital-to-PICA bypass very challenging. Furthermore, a PICA-PICA bypass would increase surgical morbidity and risk to both PICA territories. In addition, the use of dual antiplatelet therapy was not contraindicated relative to the patient’s medical history, as in the setting of subarachnoid hemorrhage due to aneurysm rupture. Lastly, after discussion of the open and endovascular options, the patient had a strong preference for endovascular therapy.

### 3:15 Operative Plan.

We planned to load the patient with dual antiplatelet therapy 1 week prior to intervention and confirm with platelet function testing. In this case, we planned to approach this left PICA aneurysm through a left radial artery approach with a 6-Fr sheath, as this would allow us to easily cannulate the left vertebral artery and establish a coaxial system. After accessing the PICA past the aneurysm, we would then deploy a 2.25 × 15–mm Silk Vista Flow Diverter across the diseased segment of the vessel.

### 3:48 Silk Vista Baby.

The Silk Vista Baby is the newest iteration of the Silk Flow Diverter. It is very low profile, designed for treatment of aneurysms in vessels 1.5–3.5 mm in diameter, and is delivered through 0.017-inch catheter. It is not currently approved for aneurysm treatment by the US FDA; thus, IRB approval for compassionate use of the device must be obtained. Initial case reports and case series demonstrate safety and show efficacy, with the consensus opinion stating that is its ideally suited for distal aneurysms.

### 4:23 Operative Video.

First, the left radial artery is accessed and a 6-Fr slender sheath is placed. The left vertebral artery is easily selected, and a Catalyst 5 distal access catheter is tracked up through the artery under roadmap guidance. An initial diagnostic angiogram is obtained with AP and lateral views, and aneurysm views are set up in preparation for device deployment—a more lateral view on the left, and AP view on the right. Next, a Synchro 10 microwire and Phenom 17 microcatheter are placed through the distal access catheter to establish a coaxial system, and the wire is navigated past the aneurysm into the lateral medullary segment. The microcatheter is then tracked past the aneurysm as well. The device is then placed in position in the distal lateral medullary segment and deployed. As the device is unsheathed, notice how easily the microcatheter tracks along the device. Here the microcatheter has reached the proximal marker on the device, with good apposition across the neck of the aneurysm. However, a run after the device is completely deployed then showed an area of focal stenosis across the proximal opening of the stent. A wire was navigated through the device past the aneurysm once again and a 4 × 7–mm TransForm Balloon was inflated across the area of stenosis under continuous fluoroscopy. A run after the angioplasty shows improved but moderate stenosis and some contrast stasis within the aneurysm. Left AP and lateral vertebral artery runs show no evidence of distal emboli or other complications.

### 6:22 Outcome.

Overall, this patient did well, was neurologically intact after the procedure, and discharged home on postprocedure day 1 on dual antiplatelet therapy. A 1-year follow-up angiogram showed complete obliteration of the aneurysm.

### 6:38 Follow-Up Angiogram.

Here, magnified lateral and AP projections are seen with no evidence of residual or recurrent aneurysm. In addition, the previously seen stenosis within the proximal segment of the device has completely resolved.

### 6:52 Conclusions.

In conclusion, the Baby Silk Vista adds another tool to the armamentarium for small-vessel aneurysms, with high rates of aneurysm occlusion.

What’s more is that this device is very low profile, and thus is suitable for distal artery access with a 0.017-inch microcatheter and can be deployed with a coaxial technique without the need for more support.

We hoped you enjoyed our video and thanks for watching.

### 7:17 References^1–7^

## Disclosures

The authors report no conflict of interest concerning the materials or methods used in this study or the findings specified in this publication.

## Author Contributions

Primary surgeon: Kan. Assistant surgeon: Lazaro, Gadgil. Editing and drafting the video and abstract: Kan, Lazaro, Vasandani, Robledo. Critically revising the work: all authors. Reviewed submitted version of the work: all authors. Approved the final version of the work on behalf of all authors: Kan. Supervision: Kan.

## Supplemental Information

### Patient Informed Consent

The necessary patient informed consent was obtained in this study.
